# “I Just Want to Be Me, Authentically”: Identity Shifting Among Racially and Ethnically Diverse Young Adults

**DOI:** 10.1007/s10964-023-01744-3

**Published:** 2023-02-15

**Authors:** Aerika Brittian Loyd, Dulce Wilkinson Westberg, LeNisha Williams, Marisha Humphries, Alan Meca, Julie C. Rodil

**Affiliations:** 1 University of California, Riverside, Riverside, CA USA; 2 University of Minnesota, Twin Cities, MN USA; 3 University of Illinois Chicago, Chicago, IL USA; 4 University of Texas at San Antonio, San Antonio, TX USA; 5 Old Dominion University, Norfolk, VA USA

## Abstract

Identity shifting represents a common but complex social, behavioral, and cognitive phenomenon. However, some forms of identity shifting originate in response to structural, institutional, and interpersonal marginalization enacted on lower status groups, such as people of color in the United States. The current study investigated ways young adults from diverse ethnic/racial groups discussed shifting to fit in with White Americans (a dominant group) in the United States and their own ethnic/racial group (a minoritized group) and elucidated self-reported motivations for shifting. Participants consisted of 764 young adults (ages = 18–23) recruited from two large public universities in the Southeast and Southwest regions of the United States. The majority of participants identified as Black/African American (41%), Asian/Asian American (27%), or Hispanic/Latinx (22%). Analysis of participants’ qualitative responses identified six types of shifts and two motivations for shifting. The shifts included: *behavioral, linguistic, cognitive, physical, food, and affect*. Motivations for shifting focused on avoiding risks and obtaining rewards. The discussion offers interpretation of the results and recommendations for future research on identity shifting.

## Introduction

Racism poses a significant challenge to positive identity development among people of color in the United States who experience discrimination at the individual, interpersonal, community, and societal level due to their race and ethnicity^1^ (Gee et al., 2012). In the United States, white supremacy and assumptions of white normativity serve as a developmental and social context that often require people of color to shift towards whiteness^2^ (Moffitt & Rogers, 2022; Spanierman, 2022). In such a context, it is common and, in some cases, necessary for individuals from racially and ethnically minoritized groups to shift or modify their identities to survive, adapt, cultivate a sense of belonging, and meet the demands and expectations of their social environment. This dynamic and multifaceted process has been described as *identity shifting* (e.g., Carr et al., 2021; Jones & Shorter-Gooden, 2003). This study examined how diverse college students engaged in outgroup (to fit in with White Americans) and ingroup (to fit in with their own ethnicity/race) identity shifting. In tandem, motivations for identity shifting were explored.

### Theoretical Foundations for Identity Shifting

Identity shifting theory and research can be traced back to notions of a differentiated self (distinguishing the knower from what is known; James, 1890) and a looking-glass self (Cooley, 1902). Seeking to understand the experiences of Black/African Americans navigating individual, interpersonal, and structural systems of racism and marginalization, DuBois (1903) & Boykin (1986) both discussed how individuals of African descent in the United States construct a differentiated consciousness to maintain a sense of self in the presence of competing social demands and racism-related threats. For DuBois, this concept was described as *double consciousness*, or knowledge of the self as authentic and understanding the self through the eyes of others (White Americans). Boykin examined this concept in three parts, navigating mainstream White America, Black culture, and being an oppressed minority (i.e., the *triple quandary*) among Black/African American children. Both perspectives emerged from a critical need to understand the experiences of Black/African Americans in the United States and to advocate for equitable treatment and policy given the historical context shaping experiences of this group today. For example, the history of enslavement, which led to a loss of indigenous cultural values, practices, and languages, laws that defined Black/African Americans in the United States as property, policies that supported economic and educational inequality (e.g., discriminatory housing practices and “separate but equal” edicts), and persistent interpersonal, societal, and legal marginalization (Parham et al., 2015).

Expanding on this theoretical and empirical legacy, identity shifting is broadly viewed as a social, behavioral, and cognitive phenomenon that has been studied under the umbrella of several related concepts, including acculturation and biculturalism (e.g., LaFromboise et al., 1993; Meca et al., 2019), code-switching (e.g., Auer, 1998; Molinsky, 2007), cultural frame switching (e.g., Hong et al., 2000; Ramírez-Esparza et al., 2006), self-monitoring (e.g., Snyder, 1979), and impression management (e.g., Leary & Kowalski, 1990). While some literature describes the general ways individuals attempt to control their self-representations (e.g., self-monitoring, impression management), other perspectives have attempted to capture the experiences of marginalized identities, such as race (e.g., code-switching) and immigration status (e.g., alternating identity styles, cultural frame switching). This study examined identity shifting related to ethnicity/race.

### Conceptualizing Identity Shifting

Here, identity shifting is defined as self-altering strategies that individuals utilize to meet the perceived demands of their social surroundings as they pertain to one’s ethnicity/race. This may include altering aspects of one’s self-presentation (e.g., mannerisms, speech) to accommodate dominant society and/or aligning the self with one’s heritage to fit in with members of one’s own ethnic/racial group (Johnson et al., 2016). Shifting may represent an adaptive response to identity-threatening experiences or expectations of discrimination (Johnson et al., 2016). In this case, shifting may serve as a coping mechanism, which minimizes the degree to which ethnic/racial biases are directed toward individuals from minoritized groups. Shifting between cultural frames may also be indicative of an identity marked by compartmentalization and conflict (Benet-Martínez et al., 2021), which may instigate psychological distress (Dickens & Chavez, 2018).

In qualitative research focusing on the experiences of Black/African American women, researchers identified behavioral and cognitive types of identity shifting (Jones & Shorter-Gooden, 2003). Women altered their behavior to transcend racial stereotypes, scanned and examined the environment for threats, denied the role of discrimination, sought support in social, religious, and cultural communities, and actively resisted identity threats. In other work also involving Black/African American women, researchers identified three types of identity shifting including awareness of shifting behaviors, adopting the *Strong Black Woman* schema (i.e., being strong and resilient despite insurmountable challenges), and expressing sensitivity to judgment from the ingroup (Johnson et al., 2016). These types of shifting appeared to reflect cognitive and behavioral themes. In work involving Latina American women, researchers also identified three types of identity shifting comprised of altering one’s speech, behaviors, and mannerisms (i.e., cultural presentation appropriateness), demonstrating language appropriateness (e.g., knowing when to speak English and Spanish), and appealing to white ideals (e.g., food, beauty, modifying an accent; Gamst et al., 2019). Finally, in research involving Black/African American and Hispanic/Latinx college students on perceptions of *acting white* (problematic accusations that represent cultural invalidations), participants described four themes that are relevant for the current study: speech/behavior, style/social preferences, cultural ideologies, and academics/success (Durkee et al., 2019). Although not seeking to investigate identity shifting specifically, this study demonstrated participants’ awareness of complex racial issues and reflected their understanding of a potential identity threat (e.g., cultural invalidations from ingroup and outgroup members) that may influence shifting around ethnicity/race.

To date, some studies elucidated how people of color, particularly Black/African Americans, shift their identities to fit in with expectations from the dominant racial group (e.g., Dickens et al., 2019; Johnson et al., 2016), and implications of this form of shifting for health and well-being (e.g., Johnson et al., 2022), less research has explored this phenomenon among other racially marginalized groups. The current study built upon previous literature by including individuals from several ethnic/racial groups (e.g., Asian/Asian American, Black/African American, Hispanic/Latinx, and Multiracial). In addition, an inductive or data-driven approach to identify themes within participants’ responses that compliment and extend previous research on identity shifting was implemented.

### When Might Identity Shifting Occur and Why?

Identity shifting can be expressed as an active or passive, and conscious or unconscious, phenomenon. In some cases, individuals may not be aware they are shifting their identities. For example, experimental work on stereotype threat (and responses to identity threat) hints at individuals’ unconscious cognitive and behavioral shifts (Steele & Aronson, 1995). The current study focused on conscious forms of identity shifting, which require knowledge that an action, thought, or behavior has or will take place.

In terms of antecedents, some research indicates awareness of social hierarchies precedes identity shifting (Gray et al., 2018). In this case, some individuals may modify or shift their language, behaviors, or self-presentation to fit their assessment of environmental threats and/or social expectations (e.g., Cole & Jacob Arriola, 2007; Lacy, 2004). This can happen in the moment as well as over time. Other research suggests changes must be within the person’s control for identity shifting to occur (e.g., Carr et al., 2021). For example, thoughts may be easier to shift than physical characteristics or language (e.g., an accent). Additionally, certain physical characteristics may be more identifiable and noticeable for some individuals (e.g., skin color, facial features, hair texture) and place them at greater risk for othering and harm (Eberhardt et al., 2006). Yet, there has been little research to explore the diverse ways people of color shift their identities based on reference group (e.g., dominant racial group vs. their own ethnic/racial group).

Further, research suggests that identity shifting is context-dependent (Carr et al., 2021). Thus, college campuses are an ideal context to study shifting because attending college may be the first place many young adults have meaningful encounters with racially diverse others (Gaither & Sommers, 2013; Solórzano et al., 2000). Although some students may have been taught to shift early in their development through family or school socialization (Umaña-Taylor & Hill, 2020), for other students, college may be the first time ethnic/racial identity shifting occurs (Chavous et al., 2018). Indeed, advances in cognition and identity development from adolescence into young adulthood allow individuals to think more deeply about shifting as well as structural inequalities and inequities that may affect their future (e.g., opportunities, interactions with peers, professors, and work; Syed & Mitchell, 2013).

Some people may engage in shifting towards the dominant group (i.e., outgroup shifting) to avoid perpetuating stereotypes and attempt to improve others’ perceptions of their group (Jones & Shorter-Gooden, 2003). For example, both *John Henryism* (Hudson et al., 2016; James, 1994) and the *Strong Black Woman* schema (e.g., Anyiwo et al., 2022; Watson & Hunter, 2016) contain elements of expending intense effort to overcome racial prejudice and social stress at the potential expense of one’s mental and physical health, as well as cultural resilience. In a university setting, outgroup shifting may be prompted by threats in the environment, including discrimination and race-related power dynamics, which manifest in the classroom through personal relationships with professors and peers (Nadal et al., 2014; Sue et al., 2009) and through university policies (Keels et al., 2017). For working college students, these experiences may also occur in the workplace (Gray et al., 2018). Considering the fundamental human needs around belonging and acceptance, there may be some perceived benefit for outgroup identity shifting, such as avoiding harmful consequences or reinforcing one’s self-worth.

Ingroup identity shifting among students of color has not been adequately explored, and there are unanswered questions around how and why individuals may shift their identities to fit in with their own ethnic/racial group. Some may engage in ingroup shifting based on the desire to maintain cultural heritage, fit in, and feel they belong with one’s group, and respect cultural norms. Others may do so to avoid accusations of acting white (Durkee et al., 2019). Indeed, the concept of intragroup marginalization captures the degree to which individuals feel detached, distanced, or even ostracized from members of their own cultural heritage group for engaging in behaviors aligned with the dominate majority group (Castillo et al., 2007).

In the current study, motivations for outgroup and ingroup shifting were expected to differ. For example, outgroup shifting may be motivated by a desire to avoid harm or gain acceptance by an outgroup in possession of societal power and privilege (e.g., Jones and Shorter-Gooden, 2003), while ingroup shifting may occur to gain a sense of belonging and avoid ingroup rejection (Tajfel & Turner, 1979). Examining outgroup and ingroup shifting within the same study allows for an important and understudied comparison between the two processes.

## Current Study

While previous literature has investigated how people of color in the United States shift their identities to fit in with the dominant racial group (i.e., White Americans), limited research has examined the dynamic tension that exists between dominant outgroup shifting and minoritized ingroup shifting. Further, few studies have explored explanations and motivations for identity shifting. To advance the identity shifting literature, the current study investigated the ways in which racially and ethnically diverse college students in the United States discussed shifting to fit in with White Americans (outgroup) or their own ethnic/racial group (ingroup) and explored participant’s self-reported motivations for shifting.

## Method

### Participants and Procedure

Participants consisted of 779 undergraduate students who were recruited from two large (each representing more than 15,000 students) public research universities in the Southeast and Southwest regions of the United States from 2019 to 2020. At the time of data collection, the student population of the Southeast university was comprised of 48.0% non-Hispanic White, 29.0% Black/African American, 8.5% Hispanic/Latinx, 6.4% Biracial/Multiracial, 4.7% Asian/Asian American, 3.0% International, 0.2% Native Hawaiian or Pacific Islander, and 0.2% American Indian/Native American. The student population of the Southwest university was comprised of 38.3% Hispanic/Latinx, 30.4% Asian/Asian American, 13.2% non-Hispanic White, 7.8% International, 5.5% Multiracial, 3% Black/African American, 1.6% Domestic unknown, 0.1% Native Hawaiian/Pacific Islander, and 0.1% American Indian/Alaskan Native. From 779 participants, 15 participants were excluded who identified as White, Anglo, European American; not Hispanic.

This resulted in a final analytic sample size of 764 participants. Participants were ages 18 to 26 (*M* = 19.80 years, *SD* = 1.98) and identified as 41% Black/African American, 27% Asian/Asian American, 22% Hispanic/Latinx, 6% Multiracial, and 4% provided an ethnic/racial group that was not listed in the demographic part of survey (including 10 Middle Eastern/Arab, 4 Egyptian, 2 South Asian, 2 Pacific Islander, 2 Palestinian, 1 Armenian, 1 Iranian, 1 Persian, 1 West Indian, 2 selected Other but did not specify). The sample included 571 Women, 184 Men, 3 Transgender, and 4 who identified with a gender not listed in the survey (2 non-binary, 1 agender, and 1 selected Other but did not specify). Most participants (84%) reported being born in the United States.

Data were collected using an online survey examining cultural adaptation among ethnic/racial minority college students and its impact on psychosocial functioning. Across both universities, students were recruited from the psychology department participant pool. Only participants who self-identified as a member of an ethnic/racial minoritized group and could read English were eligible to participate. Participants completed the online anonymous survey at their convenience. After providing informed consent and demographic information, participants completed self-report measures of identity and other indicators of psychological functioning and risk behaviors unrelated to the current study. In exchange for their participation, participants received credit toward a university research requirement. The study was approved by the Institutional Review Board at both participating universities.

For the current study, participants were presented with the following prompts: “Are there ways in which you have tailored or altered your behaviors or self-presentations when interacting with [**White Americans to appear ‘more American’/members of your own ethnic/racial group**]? Please describe.” All participants were asked to describe the reasons why they tailored or altered their behaviors or self-presentations when interacting with White Americans or their own ethnic/racial group. Participants’ descriptions of each shift were aggregated with the reason they perceived the shift to have occurred. Participants’ descriptions of tailored or altered behaviors around White Americans are referred to as “outgroup shifts” and around members of their own ethnic/racial group as “ingroup shifts”. Of the total analytic sample (*n* = 764), 458 participants (60%) did not provide an outgroup shift and 306 (40%) participants provided an outgroup shift. Five hundred and thirty-three participants (70%) did not provide an ingroup shift and 231 participants (30%) provided an ingroup shift.

### Qualitative Analysis

The authors followed recommendations for thematic qualitative analysis (Creswell, 2013; Saldaña, 2016). In the first phase of analysis, the first author reviewed all participants’ responses for content, categories, and themes. Content and categories were collapsed into common themes that were informed by published literature (e.g., Gamst et al., 2019; Jones & Shorter-Gooden, 2003). During this phase of coding, all authors reviewed the categories and discussed emerging themes. This first round of open coding informed the development of a codebook, which contained six types of shifts and two motivations for shifting. The identity shifting codebook is publicly available (Westberg & Loyd, 2023). Once the codebook was established, the second author used it to code all responses for themes on a dichotomous scale (0 = *absent*, 1 = *present*). The types of shifts were coded in a non-mutually exclusive fashion such that each response could receive a “hit” for more than one type of shift. However, since motivations for shifting were expected to be oppositional, motivations were coded in a mutually exclusive fashion such that each response could receive a “hit” for only one of the two motivations. Responses could also be coded as a “miss” for all categories. Coding was conducted at face value without inference or interpretation and was based on the entire response as opposed to a certain feature of the response (Syed & Azmitia, 2008).

To establish interrater reliability for the types of shifts and motivations, two coding teams, each consisting of two trained coders, received the codebook and a spreadsheet containing either 80 outgroup or 80 ingroup responses. Each team met weekly with the second author to resolve major coding discrepancies until coding completion. At each meeting, coders were given the chance to change or maintain discrepant codes following a group discussion. The research team found acceptable interrater reliability for the types of shifts (average κ = 0.96) and motivations for shifting (average κ = 0.84; Table 1).

**Table 1:** Types of Identity Shifts and Motives for Shifting

	Outgroup κ	Ingroup κ	Description	Example
Outgroup Shifts				
Behavioral	0.93	0.84	Participant shifted their behavior to fit in with reference group.	I used to hide academic achievement, avoid eating Chinese food, and not have other Asian friends. But I’ve gotten over it, and I don’t really care. I guess I don’t blast my GPA on a megaphone, but that’s just cause no one wants to be around people who are constantly bragging about themselves. I didn’t want to be a stereotype. Now, I really don’t care. *Outgroup Shift* I try to seem “more Mexican” by pretending (sometimes) that I can understand conversations in Spanish and seeming like I am more in touch with my Hispanics roots than I actually am. Not completely knowing Spanish is something people look down on me for, so that is probably the big one. *Ingroup Shift*
Linguistic	0.96	0.93	Participant shifted the way they speak, the content of their speech, or the language they used.	Speaking more clearly, because I would hate for them to perceive me as another ignorant black person. *Outgroup Shift* I speak in a way that they know I can understand the language. I do this to show them I am not just American. *Ingroup Shift*
Cognitive	0.88	0.70	Participant shifted the way they thought about themselves and/or the environment.	When talking to White Americans, like older people, teachers, etc. I tend to talk more “white”. The tone of my voice changes to sound more “white”. I don’t want to be negatively judged or made fun of. Growing up in a very white community, Asians were commonly judged for their “weird” cultures which has made me very self-conscious about my culture. *Outgroup Shift* I dress and wear my hair differently with members of my own ethnic group because I am able to be fearless and try new things without fearing the judgement I may get if I were to change my look with people of a different ethnicity. *Ingroup Shift*
Physical	0.95	0.94	Participant shifted their appearance, including hair, dress, or make-up.	I’ll dress more proper and elegantly to appear like I’m not less than. I don’t want to appear as less than what I am. *Outgroup Shift* I might dress in tighter clothing or have my hair out more naturally. My voice changes too, and I do my makeup a little heavier. My cousins often joke me that I’m “too white” even though I’m not white at all. So, I feel like I need to change sometimes to show them I’m still part of our ethnic community. *Ingroup Shift*
Food	0.95	0.87	Participant shifted their food selection.	If I am sure that I am going to be around White Americans during my classes which take place around lunchtime, I make sure that the food I bring to class is American food and not “weird” Filipino food. The reason I do this is so that I don’t get judged by those around me and so that I won’t be faced with questions regarding my food. *Outgroup shift* I eat more ethnic food, more spicy food and reminisce about ethnic home cooking more. More comfortable eating the things I want to eat, talk about childhood like it was all shared. *Ingroup Shift*
Affect (positive)	0.86	0.92	Participant described shifting as positive or indicated they shifted by feeling more positive.	Some White Americans have stereotypes about African Americans. Some actually believe that we aren’t capable of being intelligent or speaking eloquently. I love walking into a room of White Americans and being able to show case how educated I am and then being able to find another African American in the room and switch to the ethnic side of myself and speak African American vernacular. Although I shouldn’t have to, being able to switch between the more professional and more ethnic sides of myself so effortlessly is a beautiful thing to me. *Outgroup Shift* Yes, I am just more comfortable. I feel that way because we tend to have similar family backgrounds, so we feel more closely related. *Ingroup Shift*
Affect (negative)	0.82	1.00	Participant described shifting as negative or indicated they shifted by feeling more negative.	I alter my vocabulary or word-choice around White Americans. I become more quiet or more conscious of my behavior. I feel intimidated by white people or looked down upon when I act like my most relaxed self because they often look down upon my culture and my people. I guess I hope to “fit” better, but a part of me knows I never will. *Outgroup Shift* Yes, there has been times where I would feel too “uncultured” when I’m around people my race. *Ingroup Shift*
*Motives for Shifting*				
Risk	0.86	0.86	Shifted to avoid perceived risks (e.g., discrimination)	I have changed the way I dress and speak to appeal as a professional rather than a Latina. I dress more professionally and speak without slang words that my family and friends use. I want to ensure I have opportunities in the workplace and am not singled out as a Latina or someone who isn’t qualified for a job. *Outgroup Shift* I listen to all types of music but I certainly put on my solely hip hop, R&B, and trap playlists around members of my own race. I also use more ebonics and slang than I would normally and/or around my white colleagues and friends. I have learned to tailor myself as I was often ridiculed and accused of being “too white” and so I learned how to present an acceptable version of black. *Ingroup Shift*
Reward	0.82	0.83	Shifted to obtain perceived rewards (e.g., inclusion)	I tend to change my tone of voice and fix my posture when communicating with White Americans. I tailored/altered these behaviors because I felt that it was what was needed to be taken seriously by White Americans. *Outgroup Shift* I’ve spoken about certain traditions and cultural things in order to help me fit in more. Simply to fit in and make more friends, make people like me more, and form deeper relationships through forming similarities. *Ingroup Shift*

Qualitative examples are edited for brevity. Each type of shift was coded in a non-mutually exclusive fashion using a dichotomous 1 = present, 0 = absent coding scale. Motives were coded as mutually exclusive also using a dichotomous 1 = present, 0 = absent coding scale

The authors represent three university faculty, one postdoctoral researcher, and two graduate students. Additional members of the coding team included one graduate student, one university staff member, and one undergraduate student. Regarding ethnicity/race, members of the research team identified as Asian American (*n* = 1), Black or African American (*n* = 3), Black/Latina (*n* = 1), Hispanic (*n* = 1), Latina (*n* = 1), and White (*n* = 1). Six members identified as women and two identified as men. One member did not disclose ethnicity/race or gender demographics. Most authors identified as cisgender. Relevant for the current study, research expertise of the authors includes identity development, cultural processes, intergroup dynamics, narrative methods, qualitative methods, and educational settings.

## Results

### Types of Identity Shifting

Six types of identity shifting were identified from the analysis of participants’ responses describing the ways they have tailored or altered their behaviors or self-presentations when interacting with White Americans to appear “more American” (outgroup shifting) or to fit in with their own ethnic/racial group (ingroup shifting). The types of identity shifting included: *behavioral*, *linguistic, cognitive, physical*, *food*, and *affect*. In the following sections, the types of shifting and motivations for shifting are discussed in order from most to least frequent (Table 2).

**Table 2:** Frequency of Primary Rater’s Codes for Types of Outgroup and Ingroup Shifting and Motives

	All Participants *N* = 764		Asian/Asian American *N* = 210		Black/African American *N* = 307		Hispanic/Latinx *N* = 171		Multiracial *N* = 45	
	Outgroup	Ingroup	Outgroup	Ingroup	Outgroup	Ingroup	Outgroup	Ingroup	Outgroup	Ingroup
	*n* = 306	*n* = 231	*n* = 79	*n* = 80	*n* = 110	*n* = 64	*n* = 84	*n* = 55	*n* = 16	*n* = 15
*Type of Shift*										
Behavioral	152	147	37	57	50	33	48	38	4	7
Linguistic	161	83	30	18	62	30	50	21	11	7
Cognitive	64	47	16	19	27	10	16	13	3	4
Physical	54	51	17	24	23	18	8	5	5	2
Food	16	6	11	5	2	0	1	1	0	0
Affect (positive)	2	12	0	3	2	3	0	6	0	0
Affect (negative)	10	3	1	1	4	1	4	0	1	0
*Motive for Shift*										
Risk	137	85	32	24	53	30	37	20	6	4
Reward	109	99	30	43	44	19	22	22	9	6

*N* = number of participants; *n* = frequency of shifts. Outgroup and ingroup shifts were quantified in a non-mutually exclusive fashion such that each response could receive a “hit” for multiple categories. Thus, frequencies are reported rather than percentages to provide indication of the relevance of each type of shift for each ethnic/racial group

#### Behavioral

Behavioral shifts captured instances where participants reported changing an aspect of their behavior to fit in with the outgroup or ingroup, including diminishing or denying the self, following traditions or group expectations, changing forms of self-expression (e.g., from reserved to formal or conservative to liberal), using cultural references (e.g., jokes, knowledge), changing their interests (e.g., music, dance), purchasing items (e.g., Starbucks, Sperry’s shoes), and celebrating or downplaying their achievements.

Behavioral shifts were the most frequently reported type of shift and were similarly frequent in reference to outgroup (*n* = 152) and ingroup (*n* = 147) shifting. However, the content of behavioral shifting varied based on whether participants were referencing the outgroup or ingroup. For example, behavioral shifts around the outgroup focused mainly on adapting to mainstream white culture. As one participant (Hispanic/Latinx) stated, “I definitely feel like I have to act more ‘proper’ and educated because I am a representation of my ethnicity^3^.” Notably, across types of shifting, some participants appeared to conflate ethnicity/race with class, where some participants equated professionalism and academics with “whiteness” or “being white”, which was sometimes viewed in contrast to their own ethnic/racial group. For some participants, “acting proper” or “more professional” were common ways of shifting their identity to fit in with the outgroup.

Some participants reflected on past rather than current shifting behaviors; for example, one participant (Asian/Asian American) stated:

[I] often downplayed the presence and importance my heritage has in my life. In grade school, I adjusted my interests to fit those of my white peers. I was looking for a community where I could comfortably fit into but always felt short. Adjusting what image I presented, in the past, allowed me to get closer to my white peers.

Other participants reported actively avoiding topics they believed would elicit conflict or disagreement such as race and politics. As a case in point, one participant (Black/African American) stated, “I just try not to bring up race around them because they get super uncomfortable. I also don’t like listening to hip-hop music around them because they say the N-word and that makes me and other black people super uncomfortable.”

In contrast, behavioral shifts around the ingroup tended to focus on displaying respect and reservation by subduing one’s “American-ess” in favor of expressing one’s heritage through cultural knowledge or competencies. This was especially true for Asian/Asian American and Hispanic/Latinx participants in the sample. Several participants mentioned differences between their heritage culture (typically viewed as more reserved or traditional) and American culture (typically viewed as more uninhibited or modern). For some participants, shifting to fit in with members of their own ethnic/racial group meant behaving more respectfully (especially with elders) and being less outspoken. Many participants responded to the ingroup shifting prompt with “less American” and more “cultural/ethnic”. As one participant (Hispanic/Latinx) reported, “[I] make myself seem more cultured and not Americanized.” Another participant (Hispanic/Latinx) explained, “I try to seem ‘more Mexican’ by pretending (sometimes) that I can understand conversations in Spanish and by seeming like I am more in touch with my Hispanic roots than I actually am.” Additionally, some participants reported using cultural knowledge (e.g., humor, music, historical or popular culture facts) to fit in with members of their group.

#### Linguistic

Participants reported changing their speech or language depending on the reference group. Linguistic shifting was the next most commonly reported type of shift and was discussed more frequently in reference to outgroup shifting (*n* = 162) compared with ingroup shifting (*n* = 83). This included participants changing the way they speak (e.g., accent, tone, vernacular), the content of their speech (e.g., use of slang), and the language they use when interacting with outgroup and ingroup members. For example, several participants reported talking more “eloquently or differently”, refraining from using slang, and changing the pitch or tone of their voice when interacting with White Americans. One participant (Black/African American) responded, “I speak Ebonics at home and in professional or diverse social settings I change the way I speak—even though all dialects are linguistically equal, they are not socially equal.” Another participant (Hispanic/Latinx) reported, “I stopped speaking Spanish around white people because they would look at me weird. It caused me to lose most of my Spanish and now I struggle to speak it.” Many participants mentioned tailoring their accent, as one participant (Asian/Asian American) stated, “Each time I talked to them, I would try to hide my accent so they would not laugh at me for it.”

In comparison to outgroup shifting, linguistic ingroup shifting was mentioned less frequently. Some participants simply reported changing their language to communicate with members of their ethnic/racial group (i.e., from English to their native language). In other instances, participants reported shifting or changing their speech to fit the perceived norms and expectations of their heritage culture. As one participant (Black/African American) stated, “I try to appear cooler or more ‘hip,’ or I might try to speak differently.” Another participant (Asian/Asian American) reported, “I speak in a way that they know I can understand the language.” Similarly, one participant (Hispanic/Latinx) stated, “I purposely speak in Spanish so they will feel more comfortable.” For other participants, ingroup shifting also meant assessing the cultural environment around language expression and adjusting accordingly, as one participant (Hispanic/Latinx) reported, “[I] become more expressive and attempt conversation because being quiet is rude.” For many participants, this type of shifting also included using more slang or phrases that would primarily be understood by ingroup members.

#### Cognitive

Shifts around cognition captured changes in the way participants perceived or thought about themselves and/or the environment, without mentioning changing a behavior. This included instances when participants knew they were shifting. Some participants stated that they perceived themselves or the context differently around the outgroup or ingroup (e.g., awareness of ethnic/racial discrimination or shifting to avoid stereotypes that they are aware of). Although mentioned less frequently compared to behavioral and linguistic shifts, cognitive shifts comprised the third most frequently reported type of shift and were discussed more frequently for outgroup shifting (*n* = 64) relative to ingroup shifting (*n* = 47).

Regarding outgroup shifting, some cognitive shifts involved monitoring how one is perceived and being aware of stereotypes. As one participant (Black/African American) shared, “I try to represent myself but I also make sure I don’t fall into any stereotypes. I want to represent the black community in a positive light and be a reason to change the narrative and the misconceptions that white America has for us.” Some cognitive shifts reflected awareness of racism-related policies, practices, and ideology. This awareness and meaning making is reflected in the following outgroup shift response (Black/African American participant):

I know I have internalized many of the stigmas and stereotypes that White Americans have about Black Americans and in my response, I sometimes overcorrect for them. I want to present a positive and accurate image of what it means to be a Black American. I want to combat the stereotypes. But the irony is that in so doing, I actually perpetuate them by muting who I truly am to make White Americans comfortable. I am working on this. I want to be myself in all contexts. I don’t need to prove that I am intelligent, I am. I don’t need to prove that I am professional, I am. I just need to be me, authentically. That is the greatest revolution.

Cognitive shifts were also present in response to ingroup shifting. For some participants, cognitive ingroup shifts reflected an awareness of the importance of cultural values and norms. Other participants mentioned altering their behaviors and mannerisms specifically when they knew members of their own group would be present (reflecting an awareness of the social and cultural environment). For other participants, cognitive shifts reflected an awareness of within group discrimination and the potential for exclusion. For example, one participant (Black/African American) stated, “I didn’t like when my classmates said that I talked white so I began to use slang.” In this example, the participant was aware of cultural invalidations (accusations of acting white) and shifted their use of language accordingly. Another participant (Multiracial) shared:

Sometimes my friends tell me that I am not “Asian enough” because I am only half Chinese. It’s extremely uncomfortable for me to hear that because it makes me feel like I do not belong. Even though I speak Chinese and some of my Asian friends don’t, it is still not enough to make me feel like I belong as they always call me “whitewashed”. Even though I do not really change any of my behaviors or attitudes, when I’m with my friends I definitely get weird looks from other people especially when we are eating out at Asian restaurants because I look white instead of mixed.

Another participant (Black/African American) stated, “They (ingroup) judge you for being ‘white’, but I feel more myself around black people so I want them to like me.” One participant (Middle Eastern) simply reflected, “People from my group tend to be judgmental”.

For some participants, shifting between two or more ethnic/racial groups was perceived as contentious or stressful. As one participant (Asian/Asian American) reported, “I am constantly having to prove myself as an American and as a Korean to both communities.” Another participant (Asian/Asian American) stated, “I think there is constant pressure from several sources, such as families, the media, and other institutions for one to be heavily involved in their culture and that can be hard sometimes.” Another participant (Asian/Asian American) shared, “It’s difficult and quite confusing as a child to have two different cultures inside and outside the home.” For other participants, shifting was viewed as a challenge within their control, as one participant (Black/African American) responded, “I like to think that I have several dialects and I use them based on the context.” Another participant (Black/African American) shared:

I love walking into a room of White Americans and being able to showcase how educated I am and then being able to find another African American in the room and switch to the ethnic side of myself and speak African American vernacular. Although I shouldn’t have to, being able to switch between the more professional and more ethnic sides of myself so effortlessly is a beautiful thing to me.

#### Physical

Shifts in physical appearance consisted of ways participants discussed changing or altering their appearance (e.g., hair, dress, skin color, make-up, etc.). Physical shifts were mentioned similarly frequent among outgroup (*n* = 54) and ingroup (*n* = 51) shifting. Many participants reported altering their physical appearance to adapt or conform to white standards of beauty or to fit the cultural expectations of their ethnic/racial group. For outgroup shifting, several participants reported wearing different types of clothing, wearing clothes differently (e.g., not sagging their pants), or straightening their hair. One participant (Black/African American) reported, “I used to wear clothes like my white friends.” Another participant (Black/African American) stated, “I have changed my hair, but I change hairstyles less often to avoid questions.” Another participant (Hispanic/Latinx) reported, “Growing up, I did believe the beauty standards were straight hair and blue eyes, therefore from a young age I began burning my hair to appear less curly and more straight.” In some cases, these expectations were communicated directly from employers, as one participant (Black/African American) responded, “In the military, we are required to maintain our hair in certain ways [that] conform to White American grooming standards.”

Ingroup shifting around physical appearance tended to focus on adjusting one’s appearance to fit the cultural norms and expectations of their ethnic/racial group. For example, some participants reported dressing more “modestly” or “moderately” based on cultural expectations and customs. In other cases, participants reported changing their physical appearance to fit in with peers. As one participant (Black/African American) stated:

When I go to parties that I know will consist of people my color, I dress in brands that are popular to other African American girls. I will also try out new hairstyles such as braids or twist when I know that there will be a function that a lot of black people will be attending.

Another participant (Asian/Asian American) reported, “I conform to Korean beauty styles and fashion trends that make me more appealing to those of my culture.” Generally, participants reported changing their physical appearance to match what they perceived to be valued in the specific context based on the reference group.

#### Food

Identity shifting related to food selection also emerged as a salient theme from the analysis of participants’ responses and was mentioned more frequently in outgroup responses (*n* = 16) compared to ingroup responses (*n* = 6). When considering outgroup shifts among all participants, some participants discussed White Americans’ distaste for the food of their heritage culture. As one participant (Hispanic/Latinx) reported, “The food I tend to like many ‘white Americans’ may call it gross or just judge by the way it looks.” Similarly, another participant (Asian/Asian American) replied, “If I am going to be around White Americans during my classes which take place around lunchtime, I make sure that the food I bring to class is American food and not ‘weird’ Filipino food.” Another participant (Black/African American) responded, “I have cooked food that Americans would know instead of oxtail and pelau.” Another participant (Middle Eastern) replied, “I consume ‘American’ foods I do not like in front of White Americans.”

In contrast, ingroup shifting around food was reported less frequently by participants. Among participants who discussed ingroup shifting around food, some reported simply eating foods from a specific region or culture (e.g., Korea, Pakistan, Persian), while other participants connected food with familiarity and home. As one participant (Asian/Asian American) responded, “I eat more ethnic food, more spicy food, and reminisce about ethnic home cooking more.” For some participants, eating heritage food was important to establish ingroup connection or to avoid being excluded by the ingroup, as one participant (Asian/Asian American) replied, “[I] eat foods that I would not normally eat, to not offend family or others.” Another participant (Hispanic/Latinx) stated, “[I change my] eating habits to fit into my racial group and not come off as ‘white-washed’.”

#### Affect

Some participants reported types of shifting related to their feelings and emotions, which were coded as positive or negative affect. This theme captured discussion of positive or negative emotions related to ingroup or outgroup shifting, or when participants specified that they felt more positive or negative emotions around ingroup or outgroup shifting (i.e., not when they simply mentioned positive or negative emotions). Shifting around affect was the least commonly type of shift. Generally, instances of negative affect occurred more frequently around outgroup shifting (*n* = 10) compared to ingroup shifting (*n* = 3). For example, feeling “tense” and “anxious” were commonly mentioned within outgroup shifts. When asked how they tailor themselves to fit in with White Americans, one participant (Black/African American) simply stated, “I am more tense.” Negative affect shifts also captured when participants mentioned feeling negatively about shifting. As one participant (Hispanic/Latinx) responded, “I have been a victim of prejudice so being around White Americans makes me uncomfortable sometimes.”

In reference to ingroup shifting, participants frequently mentioned more positive emotions (*n* = 12), such as feeling more “open”, “relaxed”, “comfortable” and “free”. For example, one participant (Black/African American) stated, “I am more open about my emotions in the presence of members of my own racial group.” Another participant (Asian/Asian American) responded, “Sometimes, it is easier for me to be more open since I can relate to people in the same ethnic/racial group, for example our culture.”

### Motivations for Identity Shifting

Considering the second research question, several themes emerged from the analysis of participants’ responses for why they have tailored or altered their behaviors or self-presentations when interacting with White Americans (outgroup shifting) or members of their own ethnic/racial group (ingroup shifting). Motivation themes fell under two broad categories: *avoid risks* or *obtain rewards*. Generally, risks and rewards were reported more frequently for outgroup shifting compared to ingroup shifting (Table 2). Across types of shifting and reference group (outgroup and ingroup), a common motivation for shifting was to avoid being different and to gain a sense of belonging. In the following sections, the specific content of themes around avoiding risks and gaining rewards for outgroup shifting and ingroup shifting are discussed.

#### Avoid Risks

Frequently mentioned motivations for outgroup shifting were to avoid judgement, misunderstandings, conflict, bullying, harassment, and exclusion. Some participants engaged in outgroup shifting to manage stereotypes and circumvent racial discrimination and inequitable treatment. One student (Asian/Asian American) stated, “I don’t want to be negatively judged or made fun of. Growing up in a very white community, Asians were commonly judged for their ‘weird’ cultures which has made me very self-conscious about my culture sometimes.” Another participant (Black/African American) shared, “I usually don’t wear my hair natural or out because it is viewed as ‘unruly’. Black Americans get fired and lose their jobs due to racial discrimination against hair types.” For some participants, outgroup shifting was intended to avoid being treated as a foreigner, as one participant (Asian/Asian American) explained:

White Americans often do not understand me whenever I speak English with an accent. They tend to ask me where I’m from right away. I don’t like it when they ask me that. I try to speak as “American” as possible.

Another participant (Hispanic/Latinx) shared, “At times I feel like I need to prove that I deserve to be here, even though I was born here.” For other participants, outgroup shifting was intended to prevent racism-related physical harm and violence, as one participant (Hispanic/Latinx) stated, “I do not want to become another victim of a hate crime.” Another participant (Black/African American) replied, “I don’t want to be arrested, assaulted, degraded, or killed because I’m black”. Notably, several participants expressed a desire to avoid misunderstandings around political views that are often conflated with (mis)treatment of communities of color, policing, and government policies. For example, one participant (Black/African American) shared, “I do not want them to believe that I am anti-American for speaking on political matters that affect my racial group.” Another participant (Hispanic/Latinx) stated, “I know that I will be othered even further and that any attempts to get them to see things from my point of view will be futile.”

Regarding ingroup shifting, the desire to avoid judgement from ingroup members was also a frequently mentioned motivation. Several participants mentioned not wanting to be judged or excluded for being viewed as “too white” or “white-washed”. Among some participants, ingroup shifting was intended to avoid or prevent within group discrimination (from ingroup members) and rejection. Some participants engaged in ingroup shifting to not offend members of their own ethnic/racial group, especially when participants perceived stark differences in expectations and norms between the dominant group and their own ethnic/racial group.

#### Obtain Rewards

For outgroup shifting rewards, frequently mentioned motivations centered around the desire to fit it and be accepted. Some participants reported feeling more understood culturally and linguistically through outgroup shifting. For some participants, outgroup shifting was viewed as necessary for gaining or maintaining employment. Other participants discussed shifting was intended to overcome racial stereotypes and improve perceptions of their group. One participant (Asian/Asian American) stated, “I feel like I need to impress and break established stereotypes of society.” Another participant (Hispanic/Latinx) reflected, “I feel like I have to break their stereotype of my race.” As previously mentioned, some participants appeared to conflate ethnicity/race and class (and professionalism), as one participant (Hispanic/Latinx) shared, “I want to ensure I have opportunities in the workplace and am not singled out as a Latina or someone who isn’t qualified for a job.” Another participant (Black/African American) shared, “Not to appear more American but to appear more professional because I was in the presence of important people.” Some participants shared that outgroup shifting was relationally beneficial for making friends and ensuring more positive interactions with outgroup members. For instance, some participants perceived white individuals to be nicer and more respectful to people of color when they shifted to fit in with them.

Rewards for ingroup shifting were similar to rewards for outgroup shifting in terms of fulfilling students’ desire to fit in and belong, to gain acceptance, and develop connection with others. In many cases, participants expressed that ingroup interactions allowed them more freedom and comfortability to be themselves. Some participants shared motivations related to family and community (e.g., desire to make parents or family proud). One participant (Asian/Asian American) shared, “I don’t upset elder family members in the community, creating an opportunity for my parents to feel proud of me in a public setting.” Another participant (Asian/Asian American) expressed a desire to be an “ideal Asian son” in front of their parents and members of their own ethnic/racial group. An additional reward related to ingroup shifting was simply to uphold and maintain cultural traditions and expectations and express cultural pride. One participant (Hispanic/Latinx) shared, “To show them I’m still part of our ethnic community.”

## Discussion

Limited research has examined the diverse ways racially and ethnically minoritized individuals shift their identities around ethnicity/race to fit in with a dominant group (i.e., White Americans) in the United States and the ways they may shift their identities to fit in with their own ethnic/racial group in the same study. Even less research has investigated nuanced motivations for engaging in identity shifting around ethnicity/race. Using a qualitative design, the current study advances the literature by examining how and why individuals engage in ethnic/racial identity shifting. This approach allowed for a substantiation of themes identified in prior research and allowed for new themes and topics to emerge. Although conducted in the United States, this research holds implications for international scholarship broadly focused on understanding diversity and marginalization.

### Types of Identity Shifting

Collins (2002) stated, “Knowledge without wisdom is adequate for the powerful, but wisdom is essential to the survival of the subordinate” (p. 257). Considering this perspective, identity shifting around ethnicity/race may be essential and necessary for survival among people of color in the United States (and minoritized groups in other countries struggling to overcome persistent racial hierarchies), despite the potential impact on one’s identity. The current findings appear to support the nuances of identity shifting based on reference group and emphasize the need for future research to employ a multidimensional and multi-intentional conceptualization of identity shifting.

Collectively, the findings support prior research indicating identity shifting to be a multifaceted and multidimensional process (e.g., Gamst et al., 2019). The current study elucidated several types of identity shifting around ethnicity/race, including behavioral, linguistic, cognitive, physical appearance, food, and affect. Findings support existing literature in confirming the myriad and complex ways individuals engage in identity shifting. Findings also aligned with prior research on identity integration and bifurcation (e.g., Benet-Martínez et al., 2021; Syed, 2010), in that some participants acknowledged compartmentalizing, and others noted integrating aspects of their identity based on the reference group. While responses reflect participants’ retrospective, current, and in some cases planned activities, additional research is needed to explore how identity shifting unfolds over time.

Behavioral shifting was the most mentioned form of identity shifting in the current study and captured changes to specific behaviors based on reference group. This theme captured active attempts to make oneself more or less noticeable, following traditions, and changing one’s preferences or behaviors to meet the reference group’s expectations. Notably, this theme also captured behaviors that would signal group membership. For example, one participant mentioned drinking Starbucks in reference to outgroup shifting, which is associated with higher class in some regions (Bookman, 2013). This finding also aligns with studies investigating how people of color “perform respectability” to gain upward mobility (e.g., Landor & Barr, 2018). Since exploring participants’ meaning making around race, ethnicity, and class is beyond the scope of the current study, additional research on this topic is needed. Additionally, examining how motivations for shifting vary based on economic background may be especially fruitful, as many motivations featured a desire to enhance social standing.

The theme around linguistic identity shifting aligned with prior literature on code-switching (e.g., Durkee et al., 2019; Jones & Shorten-Gooden, 2003) and other forms of language identity shifting. Since participants were specifically asked about the ways they changed their behaviors to fit in with the dominant outgroup and their own ethnic/racial group, it is not surprising that they reported shifting their language and speech in diverse ways, ranging from what language they spoke to how they spoke (e.g., slang, accent, tone, vernacular). Future research is encouraged to compare the challenge of linguistic shifting from outgroup or ingroup across different contexts (e.g., school, home, work). Since the survey items were administered in English, and we did not ask participants for language proficiency, additional research is needed to explore this process more thoroughly in bilingual/multilingual and non-native English speakers.

The findings around cognitive identity shifting were the most nuanced identity shifts explored in the current study. Although cognitive identity shifting has been identified in prior literature (e.g., Jones & Shorten-Gooden, 2003), findings from the current study captured the dynamic tension that exists for students of color around ethnic/racial identity shifting based on reference group. Findings from the current study elucidate the complexity of participants’ thoughts about identity shifting. In some cases, participants responses appeared to reflect mixed thoughts (Dickens & Chavez, 2018) and in other cases, a sense of bicultural conflict (Benet-Martínez et al., 2021) was noted. Perhaps, when one explores what it means to be a member of multiple cultural groups, individuals become more aware of subtle cognitive shifts. Cognitive identity shifting around the outgroup also captured an awareness of racial hierarchies and critical reflection (e.g., one component of critical consciousness; Hope et al., 2020), while cognitive identity shifting around the ingroup captured awareness of intragroup marginalization (Castillo et al., 2007). Thus, it is possible identity shifting is not necessarily “good” or “bad”. Instead, the potential impact of identity shifting may be contingent on the type of identity shifting, underlying motive, subjective interpretation, and perceived distress and conflict associated with identity shifting, as well as one’s current identity structure. Future research is necessary to more carefully capture the multidimensionality of identity shifting and to determine whether certain aspects, as well as certain motives and conditions, are associated with psychosocial functioning.

The inductive approach used in the present study allowed the authors to discern themes in unique ways (Dooley et al., 2020). For example, identity shifting around physical appearance, which referenced attempts to alter one’s dress, hair, make-up, and in some cases, skin color, was distinct from behavioral shifting, more broadly. Future research should further explore identity shifting around physical appearance to distinguish between normative identity exploration that is common among youth and young adults (Arnett, 2000) and physical shifts that occur due to institutional and structural bias (Jones & Shorten-Gooden, 2003). In addition, future research should explore gender differences in physical appearance shifting, as past work indicates women of color may experience unique forms of gendered racial bias and discrimination (e.g., Moody & Lewis, 2019; Thomas et al., 2008).

Findings regarding identity shifting around food also aligned with prior literature. For example, in exploring identity shifting in Latina American women, researchers similarly identified shifting food preferences to reflect white ideals (Gamst et al., 2019). The current study reveals that shifting around food preferences occurs for potentially different reasons based on reference group. For example, some participants in the current study reported consuming foods, which they do not prefer, to be accepted by outgroup members (e.g., appealing to white ideals) or avoiding cultural foods in the presence of White Americans. In contrast, other participants reported eating cultural foods based on preference and connection to the group. Identity shifting around food was also reported as a strategy to avoid intragroup marginalization and increase connection with group members. Overall, shifting around food occurred for myriad and complex reasons.

Findings around affect aligned with prior research on biculturalism and bicultural identity integration (Benet-Martínez et al., 2021), such that participants reported positive and negative feelings and emotions around ethnic/racial identity shifting. This was the case for both outgroup shifting and ingroup shifting, though outgroup shifting tended to be more negative (e.g., tense, anxious) while ingroup shifting tended to be more positive (e.g., free, relaxed). Additional research is needed to explore specific emotions around identity shifting, and importantly, the potential relationship between identity shifting, emotions (including emotional regulation), health, and well-being. Doing so may shed better light on the potential connection between identity shifting and overall adjustment among minoritized individuals and groups.

### Motivations for Identity Shifting

Motivations appeared to be both negative and positive in reference to outgroup and ingroup shifting. Across both outgroup and ingroup shifting, participants reported a desire to belong, fit in, and gain acceptance. Thus, shifting may be an attempt to fulfill a fundamental human need for acceptance and connection (Deci & Ryan, 2012). Furthermore, common risks included the fear of being othered or experiencing discrimination, potentially the need to avoid harm (e.g., emotional, social, psychological, and physical).

Although motivation around risks and rewards for both outgroup and ingroup shifting were observed, the content of participants’ responses did vary by outgroup and ingroup. For example, responses related to risks associated with not shifting to fit in with the outgroup (perceived fears) included foreigner objectification (e.g., Armenta et al., 2013) and physical harm. For ingroup shifting, participants were wary of cultural invalidations from their own group including intragroup marginalization and accusations of “acting white”. Although motivation was coded as mutually exclusive to differentiate when participants shifted to avoid something versus to gain something, future research should consider that motivations can have multiple intentions and seek to further explore the phenomenological meaning behind identity shifting. Essentially, motivations can be “and” rather than “or” (Overton, 2010).

Regarding rewards, outgroup shifting appeared to confer more benefits in educational and professional spaces, which, could be argued, were primarily viewed as “white spaces” by participants. Thus, it is possible shifting was perceived as necessary for educational and professional attainment or improving the perception of the group. One could argue these rewards are linked to risks. In contrast, rewards for ingroup shifting aligned with research on cultural values around family relationships (e.g., filial piety and familism; Meca et al., 2022) and general tendency for youth to gravitate towards biculturalism (Huynh et al., 2011; Schwartz et al., 2019). Consistent with recommendations focused on how bicultural youth engage in bicultural negotiation strategies (Meca et al., 2019; West et al., 2017), future research might explore the ways students of color maintain and sustain cultural values and heritage through shifting in the context of identity threats, and at the same time, explore how cultural identity development informs identity shifting.

### Limitations and Future Directions

This study advances the literature around identity shifting and carries several significant strengths (e.g., data collected from participants in two regions, two universities, and sample size), but it is not without limitations. The first limitation is related to measurement. Participants were asked about the ways in which they have “tailored or altered their behaviors or self-presentations”, which may have elicited responses about behaviors and unintentionally reduced other forms of identity shifting (e.g., cognitive, affect). Said differently, participants may have been primed to think about behaviors. However, since participants in the current study spontaneously mentioned other types of identity shifting (e.g., linguistic, food), future research should consider other ways to creatively examine identity shifting that would elicit more non-behavioral types of shifting. In addition, the retrospective design did not allow the study to capture forms of identity shifting in the moment. Thus, future research might consider that individuals are constantly examining the social environment and considering how to modify or adapt to fit the social demands. Data collected at multiple time points (e.g., experience sampling methods) and multiple methods (e.g., quantitative and qualitative) will allow researchers to better understand how identity shifting happens in the moment and over time, as well as the implications of identity shifting for health and well-being. While beyond the scope of the current study and data collected, additional research is needed to understand the nature and consequences of identity shifting for minoritized groups that were not examined specifically in the current study, including immigrants and bi/multilinguals. Furthermore, given the findings of the current study, future research should consider exploring the diversity of experiences within racial and ethnic groups.

Second, additional research is needed to explore participants’ phenomenological understanding of several complex concepts mentioned in the current study (i.e., whiteness, class). Although beyond the scope of the current study and limited by the data, there were several instances in which participants responses appeared to conflate race, class, and space (e.g., education and work were viewed as primarily white and affluent spaces). Future research should further examine how these ideals are perpetuated and adopted, and how culturally diverse individuals make meaning of these concepts and spaces (e.g., Cooper et al., 2022; Seaton, 2022). While this study focused on the experiences of individuals and offers unique insights into the diverse ways individuals navigate their social environment, future research should also explore contexts and structures that may prompt individuals and groups to engage in identity shifting. Additionally, the current study did not examine specific contexts where identity shifting may occur (e.g., peer, romantic partners, work, school, home) or specifically ask participants to identify where shifting may be occurring. It is possible specific forms of identity shifting occur primarily in school and work contexts or with specific groups (e.g., peers, family members, coworkers), and motivations for shifting in these contexts may vary.

Prior research has linked identity shifting to mental and behavioral health (Johnson et al., 2022; Durkee & Gómez, 2022). Although the data appear to hint at the potential for social and psychological outcomes (e.g., sense of belonging, acceptance, rejection), it was beyond the scope of the current study to examine the effect of identity shifting on other indicators of health and well-being. Moreover, there appeared to be some important variation in the content of shifts based on ethnicity/race and certain types of shifts may function differently based on ethnicity/race. However, the disproportionate sample sizes by themes did not allow for meaningful statistical comparisons across groups. Additional work is needed to disentangle how these shifts may relate differentially with mental health and other indicators of well-being based on ethnicity/race.

Based on previous research, mentions of identity shifting around name changes were expected. Research suggests that students may change their names to avoid mispronunciations and microaggressions (Kohli & Solórzano, 2012; Moore et al., 2020). However, changing one’s name was only discussed once and thus did not constitute a salient theme in the current study (see Westberg & Loyd, 2023). Future research might explore the frequency of this practice as a form of identity shifting and examine the potential impact of name changes on health and well-being among individuals from culturally marginalized groups.

Notably, several participants (approximately 40%) reported not engaging in shifting behaviors and some provided a response, which introduces another avenue to future study. Future research should explore non-shifting as a potential act of resistance or critical awareness/consciousness action that can also be expressed multidimensionally. Studies should also examine implications of non-shifting for health and well-being in racially and ethnically minoritized individuals.

### Implications for Practice and Policy

The salience of ethnicity/race was evident in participants’ responses in the current study. Major shifts in the United States and globally around issues of diversity, equity, and inclusion since 2020 have led many institutions and organizations to address the racial climate. For universities, creating campuses of acceptance requires a re-imaging of educational spaces that includes education, training, and professional development for everyone in the ecological system. For instance, universities could use an equity assessment and evaluation process that assesses the conditions for institutions to implement sustainable change (e.g., Byrd, 2019; Harris & Bensimon, 2007). Education and training can allow for faculty, students, and staff to co-create a campus community that supports all students’ ethnic/racial identities. Based on the current study, training and education should explicitly seek to address and dismantle the reinforcement of whiteness as the standard to eliminate the pressure for minoritized individuals to shift. At the state and government level, polices aimed at reducing discrimination are also needed. Senate Bill 188 in California prohibits discriminatory actions based on hair texture and hairstyles; some have argued that a federal policy in the United States is necessary (Donahoo, 2021). Rather than simply placing burden on individuals to shift, such societal and structural changes are needed to reduce the potential for biased actions and discrimination to occur.

## Conclusion

Future research should move beyond the tendency to view identity shifting as inherently or purely adaptive or maladaptive. Identity shifting may be positive for career advancement AND negative for one’s mental health or sense of positive well-being. Identity shifting can promote a sense of belonging AND internal conflict. Moreover, these effects are likely to vary as a function of a multitude of factors, including motives for shifting, perceptions of shifting, and ones’ own cultural identity configuration. That said, whereas expectations to shift one’s behaviors and mannerisms to fit various contexts (e.g., home and work) may be reasonable for adults compared to children, it is critical to note that societal and structural pressures to shift one’s identity (e.g., hair, skin color) to appeal to a dominant social group are unreasonable and perpetuate a racial hierarchy. Thus, future scholarship should continue to explore under what conditions identity shifting may be necessary, adaptive, and forced, and the implications of identity shifting for identity development, health, well-being, as well as educational and professional endeavors. In future studies, this should be done using a variety of methods to better ascertain the bounds by which shifting relates to these outcomes. Importantly, policy changes are needed to ensure diverse individuals and groups can authentically be themselves.

## Footnotes

1 Race and ethnicity represent unique but overlapping, multidimensional constructs that are defined by shared processes, outcomes, and experiences that are not always able to be disentangled (Atkin et al., 2022; Cokley, 2007). In the current paper, the authors employ the combined term ethnic/racial.

2 For an in-depth discussion on how diverse European-descend groups became “white” in the United States, see too McIntosh (1997) & Waters (1990).

3 Qualitative responses were edited for spelling and brevity. Participants’ non/capitalization of ethnic/racial terms was retained.
